# Rethinking Preoperative Risk Evaluation: How Well Does EuroSCORE II Predict Long-Term Mortality After Cardiac Surgery?—A Single-Centre Retrospective Analysis

**DOI:** 10.3390/jcm15020837

**Published:** 2026-01-20

**Authors:** Andreas Koköfer, Lukas Simon Fischer, Bernhard Wernly, Daniel Dankl, Crispiana Cozowicz, Elke Boxhammer, Richard Rezar, Christian Dinges, Jan Waskowski, Niklas Rodemund

**Affiliations:** 1 Department of Anesthesiology, Perioperative Medicine and Intensive Care Medicine, Paracelsus Medical University, 5020 Salzburg, Austria; 2 First Department of Medicine, Paracelsus Medical University, 5020 Salzburg, Austria; 3 Center for Public Health and Healthcare Research, Paracelsus Medical University, 5020 Salzburg, Austria; 4 Department of Internal Medicine II, Cardiology, Intensive Care Medicine & Emergency Department, Paracelsus Medical University, 5020 Salzburg, Austria; 5 Department of Cardiac Surgery, Paracelsus Medical University, 5020 Salzburg, Austria; 6 Department of Intensive Care Medicine, Inselspital, Bern University Hospital, University of Bern, 3012 Bern, Switzerland

**Keywords:** EuroSCORE II, long-term mortality, cardiac surgery, cardiopulmonary bypass, risk stratification

## Abstract

**Objectives:** EuroSCORE II is widely used to predict perioperative and 30-day mortality in cardiac surgery, yet data on its ability to predict long-term outcomes remain limited. This study investigates whether EuroSCORE II is associated with one-year and long-term mortality in a heterogeneous population undergoing major cardiac surgery with cardiopulmonary bypass. **Methods:** A retrospective cohort study was conducted including 2179 patients who underwent elective or urgent cardiac surgery with cardiopulmonary bypass between 2017 and 2021 at the University Hospital Salzburg. Data were extracted from the Salzburg Intensive Care database (SICdb) and supplemented with mortality information from Statistik Austria. EuroSCORE II values were compared between survivors and non-survivors. Kaplan–Meier analyses, Cox regression and logistic regression with ROC analysis were performed to evaluate the predictive association of EuroSCORE II with mortality. **Results:** EuroSCORE II was significantly higher in patients who died within one year and in those who died during a mean follow-up period of 1152.67 ± 521.39 days. Patients who survived at least one year had a median EuroSCORE II of 2.2, whereas those who died within one year had a median of 7.0. Cox regression demonstrated a hazard ratio of 1.062 for one-year mortality and 1.058 for long-term mortality. Kaplan–Meier curves showed significantly reduced survival with increasing EuroSCORE II quartiles. Logistic regression for one-year mortality yielded an AUC of 0.773, indicating good discriminative ability. **Conclusions:** EuroSCORE II is significantly associated with long-term mortality after major cardiac surgery, demonstrating good discriminatory performance. These findings support its potential utility not only as a short-term but also as a long-term prognostic indicator in cardiac surgery populations.

## 1. Introduction

Accurate and comprehensive preoperative risk assessment is an indispensable component of modern cardiac surgery. It supports informed surgical decision making, guides communication between clinicians and patients, and serves as a cornerstone of institutional quality management [1,2]. Over the past decades, continuous improvements in surgical technique, perioperative care, and postoperative management have reduced operative mortality, intensifying the demand for risk prediction tools that accurately reflect contemporary clinical realities [3]. Among these tools, the European System for Cardiac Operative Risk Evaluation (EuroSCORE) has played a pivotal role since its introduction in 1999 [4,5]. Its successor, EuroSCORE II, published in 2012, was developed in response to increasing concerns that the original model overpredicted mortality as surgical outcomes improved [6]. EuroSCORE II incorporated revised and newly relevant risk variables, including updated classifications for symptomatic cardiac disease, urgency of surgery and renal dysfunction. Despite its widespread adoption for in-hospital and 30-day mortality prediction [7,8,9], the extent to which EuroSCORE II predicts long-term survival remains insufficiently investigated.

In Austria, EuroSCORE II is a mandatory quality-assurance measure, embedded within the national cardiac surgery registry [10]. However, validation of EuroSCORE II specifically for long-term risk prediction has not been performed on a national or to that extent international level. With the introduction of the Salzburg Intensive Care database (SICdb) [11,12], together with data from Austria’s Federal Statistical Office, new possibilities for retrospective long-term survival analyses have emerged.

This study therefore aims to investigate whether EuroSCORE II values collected preoperatively at an Austrian tertiary cardiac surgery centre are associated with one-year and long-term mortality. The analysis includes survival modelling using Cox regression, discriminative assessment using receiver operating characteristic (ROC) curves and detailed survival stratification through Kaplan–Meier curves.

## 2. Materials and Methods

### 2.1. Study Design and Population

This retrospective single-centre study included all adult patients who underwent elective or urgent cardiac surgery requiring cardiopulmonary bypass (CPB) at the University Hospital Salzburg between 2017 and 2021. Data were extracted from SICdb, which aggregates data from the hospital ICU data management system (PDMS) iMDsoft MetaVision ICU (iMDsoft, Needham, MA, USA) and the electronic health record (EHR) ORBIS (DH Healthcare GmbH, Bonn, Germany). SICdb (Version 1.0.6) was queried in May 2023, and entries up to and including December 2021 were evaluated. Data on long-term mortality are currently not reported in SICdb. Therefore, those were collected from Austria’s Federal Statistical Office (Statistik Austria, German: Bundesanstalt Statistik Österreich). Data was accessed in January 2023. All data are fully anonymized as defined by the European General Data Protection Regulation [13]. Ethical approval for the use of SICdb was granted by the State Ethics Commission of Salzburg (EK Nr: 1115/2021).

A total of 2179 patients were included in the overall cohort. For one-year survival analyses, patients with observation periods shorter than 365 days were excluded, leaving 1979 patients for analysis.

### 2.2. Variables

Patient demographic variables, comorbidities, preoperative medications, laboratory values, operative urgency and procedure types were extracted. Surgical procedures were categorized into predefined procedural groups. Complex combined procedures included cases with multiple cardiac interventions, encompassing aortic procedures. Due to the retrospective nature of the dataset, detailed intraoperative information such as specific aortic arch replacement strategies were not consistently available and therefore could not be analyzed. Furthermore, the relatively small number of patients undergoing isolated or complex aortic/arch procedures resulted in limited event counts within these subgroups, precluding meaningful stratified analyses by surgical technique. EuroSCORE II values were taken directly from SICdb. Mortality within one year and cumulative mortality during long-term follow-up were the primary outcomes.

### 2.3. Statistical Analysis

Distributional characteristics of numerical variables were assessed prior to analysis. As several key variables, including EuroSCORE II itself, did not follow a normal distribution, data are reported using medians and interquartile ranges where appropriate. Accordingly, non-parametric statistical tests were applied for group comparisons. Metric variables were summarized using means and medians, along with standard deviations (SD) and interquartile ranges (IQR), as appropriate. Categorical variables were expressed as frequencies and percentages. EuroSCORE II values were compared between survivors and non-survivors using appropriate non-parametric comparisons. Kaplan–Meier curves were generated with EuroSCORE II grouped by quartiles and percentiles.

Cox proportional hazards regression assessed the association between EuroSCORE II and time-to-death. Logistic regression evaluated the ability of EuroSCORE II to classify one-year mortality, complemented by ROC curve analysis with 10-fold cross-validation repeated 500 times. Area under the ROC curves (AUCs) with 95% confidence intervals were calculated. A significance level of *p* < 0.05 was used for all tests.

## 3. Results

### 3.1. Patient Population

The cohort consisted of 2179 patients with a mean age of 67.15 ± 10.95 years; 26.98% were female. Hypertension was present in 65.4%, diabetes in 20.65%, chronic lung disease in 9.45%, and renal dysfunction in 16.52%. Chronic renal replacement therapy was required preoperatively in 5.19% (Table 1 presents the principal demographic and clinical characteristics recorded at ICU admission). Mean cardiopulmonary bypass time was 124 ± 65.69 min, and mean cross-clamp time was 74.06 ± 41.99 min. Table 2 displays the distribution of the various surgical procedures. Urgency categories included 64.57% elective, 26.57% urgent, 7.89% emergency and 0.96% life-threatening procedures. EuroSCORE II values ranged from 0.5 to 65.0, with a median of 2.3 (IQR 3.7).

### 3.2. One-Year Mortality

**Figure 1:**
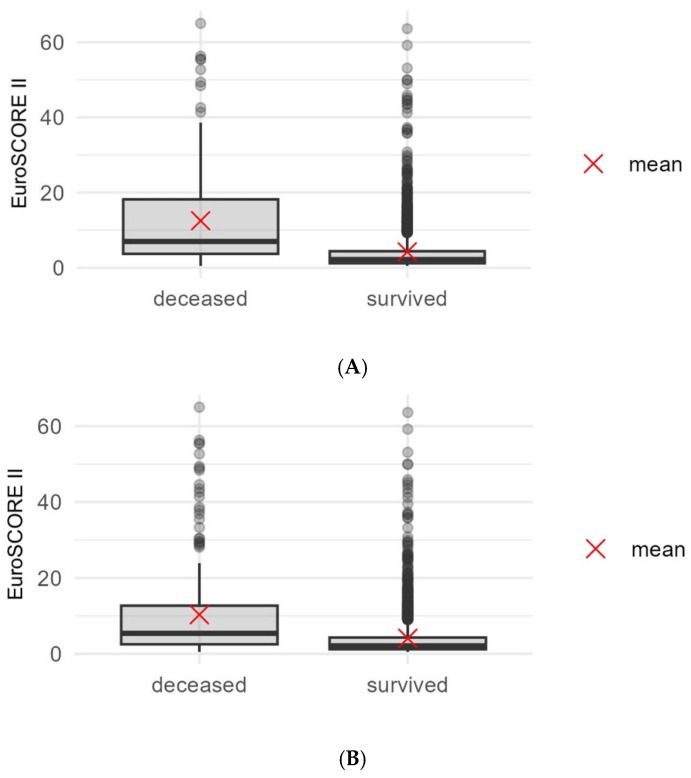
(**A**): Boxplot showing the difference in EuroSCORE II between patients surviving and being deceased within the first year. (**B**): Boxplot showing the difference in EuroSCORE II between patients surviving and being deceased in the long-term observation.

Within one year after surgery, 149 of the total 2179 patients (6.84%) had died (Table 3). Patients who survived at least one year had a EuroSCORE II ranging from 0.5 to 63.6, with a median of 2.2 (IQR 3.2). Patients who died within one year had markedly higher EuroSCORE II values, with a median of 7.0 (IQR 14.5) (Figure 1A). This difference was statistically significant (*p* < 0.001). Kaplan–Meier analysis demonstrated stratified survival across EuroSCORE II quartiles, with one-year mortality increasing progressively from the lowest to the highest quartile (graphical results not displayed). The lowest quartile (≤1.2) showed 1.65% mortality, while the highest quartile (>4.9) showed 17.26% mortality (Table 4). Cox regression showed that each 1% increase in EuroSCORE II was associated with a 6.2% increase in the risk of death within one year (HR 1.062; 95% CI 1.052–1.072; *p* < 0.001).

### 3.3. Long-Term Mortality

**Figure 2:**
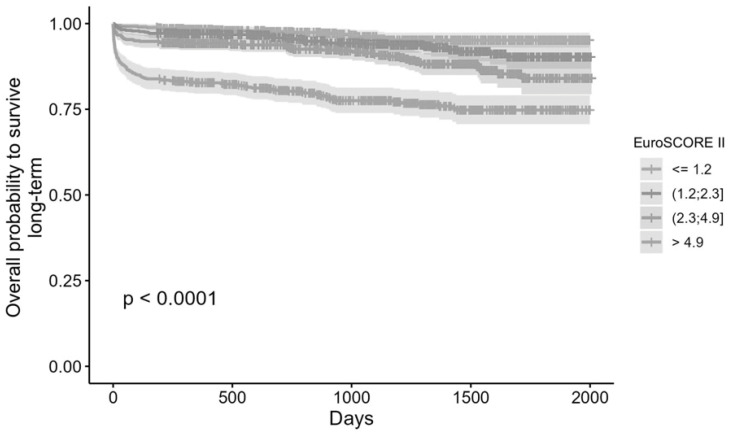
Kaplan–Meier survival curve of all patients during the observed time. Patients are separated into quartiles according to the reported EuroSCORE II.

Over a mean observation period of 1152.67 ± 521.39 days, 231 patients (10.6%) died. Survivors had a median EuroSCORE II of 2.1 (IQR 3.1), whereas non-survivors had a median of 5.4 (IQR 10.2). The difference was again significant (*p* < 0.001) (Figure 1B). Again Kaplan–Meier curves based on quartiles and percentiles consistently showed significantly reduced survival in higher EuroSCORE II strata. Kaplan–Meier curves stratified by quartiles and percentiles demonstrated significant separation (*p* < 0.001) (Figure 2). The highest percentile group (>10.92) exhibited 29.82% mortality, compared with 3.79% in the lowest percentile group (0.5–0.8). Increased EuroSCORE II was significantly associated with an increased long-term mortality (*p* < 0.001) (Table 4). Cox regression for long-term mortality demonstrated a hazard ratio of 1.058 per 1% EuroSCORE II increase (95% CI 1.049–1.067; *p* < 0.001).

### 3.4. Discriminative Ability for One-Year Survival

**Figure 3:**
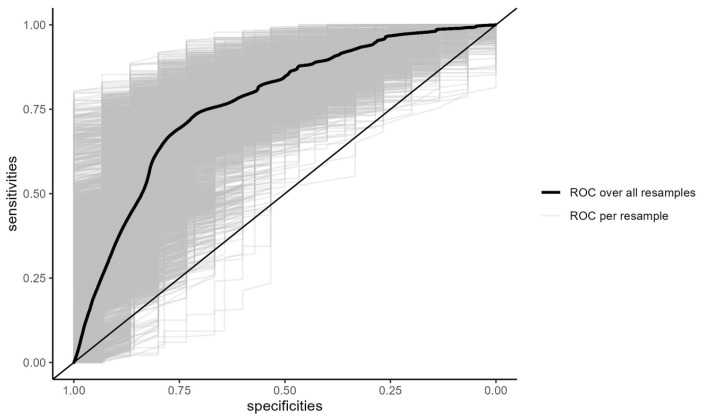
Receiver operating characteristic curve (ROC curve) of logistic regression of patients who died within the first year. The AUC of the ROC curve is 0.773 (95% CI 0.646–0.879), indicating good discriminative power.

Logistic regression with optimal thresholding at 6% probability yielded a sensitivity of 0.730, specificity of 0.712, and accuracy of 0.714. ROC analysis produced an AUC of 0.773 (95% CI 0.646–0.879), indicating good discriminative power (Figure 3).

## 4. Discussion

This study is the first Austrian single-centre analysis to evaluate EuroSCORE II as a predictor of long-term mortality in a large heterogeneous population undergoing major cardiac surgery. The results demonstrate a strong and statistically significant association between higher EuroSCORE II values and both one-year and long-term mortality. Importantly, EuroSCORE II also predicted mortality across a mean follow-up period exceeding three years. Long-term non-survivors had more than double the median score of survivors (5.4 vs. 2.1), and mortality increased steadily across quartiles and percentiles. The long-term hazard ratio (HR 1.058) nearly mirrored the one-year hazard ratio, suggesting that the variables included in EuroSCORE II reflect underlying physiological vulnerability and comorbidity burden. This vulnerability continues to influence mortality risk well after the postoperative phase. Thus, EuroSCORE II, although not designed for long-term use, provides valuable prognostic information about patient survival beyond 365 days. The primary objective of this study was to evaluate EuroSCORE II as a composite preoperative risk score rather than to re-identify individual predictors of mortality. While univariate analyses of demographic and clinical variables may provide complementary insights, such analyses were beyond the predefined scope of this study and may be more appropriately addressed in future investigations.

Our findings are consistent with existing studies reporting that EuroSCORE II, despite being calibrated for early mortality, carries prognostic information extending beyond the immediate perioperative period [14]. Barili et al. previously demonstrated declining yet significant discriminative performance for one-year and five-year mortality, reporting AUCs of 0.77 and 0.73, respectively, which align closely with the AUC of 0.773 in the present study. Similarly, Ozcan et al. reported good predictive accuracy for one-year and long-term mortality following CABG, while comparing EuroSCORE II with a new self-developed score [15]. Fleet et al. analyzed the predictive ability of EuroSCORE II in long-term mortality in a population having had CABG [16], while Toumpoulis et al. analyzed a cohort with valve surgery only [17]. However, both studies focus on a single type of cardiac surgery only and are further limited by relatively small case numbers. Very recently Bradbury et al. used EuroSCORE II even in a cohort of patients having coronary artery disease (CAD), but only 65% having CABG surgery, to predict long-term mortality. They calculated the score for each patient (also for the non-surgical patients) and defined a threshold of ≥2% to be high risk. The primary endpoint was a composite of major adverse cardiovascular events (MACEs). Ultimately they found that the EroSCORE II may also be suitable in such a non-surgical scenario [18]. However, so far there is not much evidence supporting the use of EuroSCORE II for such a non-surgical setting, nor is there any calibration [19].

However, the hazard ratios observed in this study (HR 1.062 for one-year and HR 1.058 for long-term mortality) substantiate the strong increase in risk associated with increasing EuroSCORE II values. This strong monotonic relationship was observed across all quartiles in the Kaplan–Meier analyses and highlights the clinical relevance of this association. Still, the model’s calibration for long-term prediction remains uncertain. The long-term performance of EuroSCORE II likely depends on how closely early perioperative risk factors reflect overall long-term patient vulnerability.

## 5. Limitations

The retrospective design limits causal inference, and the single-centre nature of the study restricts generalisability. Manual entry of EuroSCORE II into the dataset introduces potential data entry inaccuracies. Furthermore, patients treated at this centre but dying abroad may not have been captured in Statistik Austria records. Subgroup analyses by procedure type or isolated comorbidities were not performed due to insufficient event numbers per subgroup, potentially obscuring differential predictive performance across patient categories. A further limitation is that part of the study period overlapped with the COVID-19 pandemic, during which perioperative care pathways, case selection, and healthcare system constraints may have differed from routine practice and could have influenced clinical outcomes. Additionally, detailed data on perioperative and long-term glycemic control, including HbA1c levels, insulin therapy, and postoperative glucose variability, were not consistently available in this retrospective dataset and therefore could also not be reliably incorporated into the analysis. Nonetheless, the size of the cohort and consistent results across statistical methods strengthen our overall conclusions.

## 6. Conclusions

EuroSCORE II is significantly associated with both one-year and long-term mortality following major cardiac surgery requiring cardiopulmonary bypass. Higher EuroSCORE II values correspond to substantially increased postoperative mortality risk, and the score demonstrates good discriminative ability for long-term survival. These findings support the utility of EuroSCORE II not only as a perioperative tool but also as a long-term prognostic marker, suggesting potential value in its incorporation into extended postoperative risk stratification, therapy planning and long-term follow-up.

## Figures and Tables

**Table 1:** Demographic and clinical characteristics recorded at ICU admission; CRRT: chronic renal replacement therapy, CPB: cardiopulmonary bypass, SAPS3: Simplified Acute Physiology Score 3; KDIGO AKI: Kidney Disease Improving Global Outcomes Acute Kidney Injury.

	N (Percent)/Value (SD)
Age on admission (mean years)	67.15 (±10.95)
Female	588 (26.98%)
Height on admission (mean cm)	170.72 (±9.63)
Weight on admission (mean kg)	80.52 (±18.73)
Precondition arterial hypertension	1425 (65.4%)
Precondition diabetes	450 (20.65%)
Precondition lung disease	206 (9.45%)
Precondition renal dysfunction	360 (16.52%)
Heart surgery CPB time (min)	124 (±65.69)
Heart surgery cross-clamp time (min)	74.06 (±41.99)
SAPS3 on admission	43.81 (±11.13)
Creatinine baseline (mg/dL)	0.92 (±0.5)
Creatinine max. within 168 h (mg/dL)	1.21 (±0.8)
Acute Kidney Injury KDIGO class within 168 h	
0	816 (37.45%)
1	585 (26.85%)
2	690 (31.67%)
3	88 (4.04%)
CRRT during ICU stay	113 (5.19%)
Lactate max. on day of surgery (mmol/L)	3.43 (±1.8)
Lactate max. on day 1 after surgery (mmol/L)	2.04 (±1.51)
Norepinephrine on day of surgery (μg/kg/min)	0.08 (±0.08)
Norepinephrine on day 1 after surgery (μg/kg/min)	0.09 (±0.12)
Hs TnT on day of surgery (ng/mL)	1495.95 (±2117.27)
Hs TnT on day 1 after surgery (ng/mL)	1148.15 (±2313.97)

**Table 2:** Distribution of the various surgical procedures: CABG: coronary artery bypass grafting, AVR: aortic valve replacement, complex combined procedures and others: thoracic aortic dissection/rare interventions, e.g., for myxoma.

Procedure	N (Percent)
CABG (isolated)	785 (36.03%)
CABG (not known if in combination with other procedures)	121 (5.55%)
AVR (isolated)	23 (1.06%)
AVR (not known if in combination with other procedures)	33 (1.51%)
Combined procedures	304 (13.95%)
Neither valve nor CABG	114 (5.23%)
Neither CABG nor AVR (not known if another valve)	52 (2.39%)
Valve procedures (isolated) other than AVR	246 (11.29%)
Valves other than +AVR	501 (22.99%)

**Table 3:** Basic mortality data.

	N (Percent)/Value (SD)
Patients alive after one year	2030 (93.16%)
Patients survived + observed for at least one-year	1979 (90.82%)
Survival time of deceased patients (days)	49.6 (±72.59)
EuroSCORE II	4.74 (±7.14)
Observation time (days)	1152.67 (±521.39)
In-hospital survival	2079 (95.41%)

**Table 4:** Percentage and total number of patients per quartile of patients deceased in each EuroSCORE II quartile within one year and long term, respectively.

EuroSCORE II Quartiles	Total	Deceased (One Year)	Deceased (Long Term)
<1.2	547	9 (1.65%)	20 (3.66%)
(1.2; 2.3]	560	17 (3.04%)	36 (6.43%)
(2.3; 4.9]	539	31 (5.75%)	56 (10.39%)
>4.9	533	92 (17.26%)	119 (22.33%)

## Data Availability

The SICdb dataset is publicly available on PhysioNet (Refs. [11,12]). However, contributor-approved access is currently in place.
